# Verification of negative pressure box for preventing severe acute respiratory syndrome coronavirus 2 (SARS‐CoV‐2) transmission during upper gastrointestinal endoscopy

**DOI:** 10.1002/jgh3.12595

**Published:** 2021-06-14

**Authors:** Hideki Kobara, Noriko Nishiyama, Haruo Oba, Taichi Nagatomi, Naoya Tada, Shintaro Fujihara, Tsutomu Masaki

**Affiliations:** ^1^ Department of Gastroenterology and Neurology, Faculty of Medicine Kagawa University Kita Japan; ^2^ Department of Engineering and Design Kagawa University Kita Japan; ^3^ Center for Industrial‐Academic Partnership and Intellectual Property Kagawa University Kita Japan

**Keywords:** endoscopy: upper gastrointestinal, gastrointestinal infections, gastroenterology, protective barrier enclosures, virus transmission

## Abstract

During the COVID‐19 pandemic era, multiple infection prevention and control measures to reduce the risk of SARS‐CoV‐2 transmission may be required in upper gastrointestinal endoscopy (UGI). We herein studied the verification tests of Endo barrier, which is one of the protective barrier enclosures. Endo barrier may be an alternative for minimizing SARS‐CoV‐2 transmission during UGI.

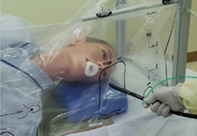

During the COVID‐19 pandemic era, multiple infection prevention and control measures to reduce the risk of SARS‐CoV‐2 transmission may be required in upper gastrointestinal endoscopy (UGI) because it is an aerosol‐generating procedure.^1^ While the use of standard personal protective equipment is recommended,^2^, ^3^ several protective barrier enclosures for blocking aerosol dispersal from patients are also under investigation. The U.S. Food and Drug Administration warns that the use of protective barrier enclosures without negative pressure may pose increased health risks during tracheal intubation in COVID‐19 patients.^4^ We therefore developed a patient‐covering negative pressure box model for UGI that uses disposable vinyl bags.^5^ Problematic issues included assembly by hand and materials not being readily available. On the basis of our concept model, the Endo barrier® (EB) (Okura Industrial Co. Ltd., Kagawa, Japan) (Fig. 1a,b) was produced to protect both patients and healthcare providers from virus transmission.

**Figure 1:**
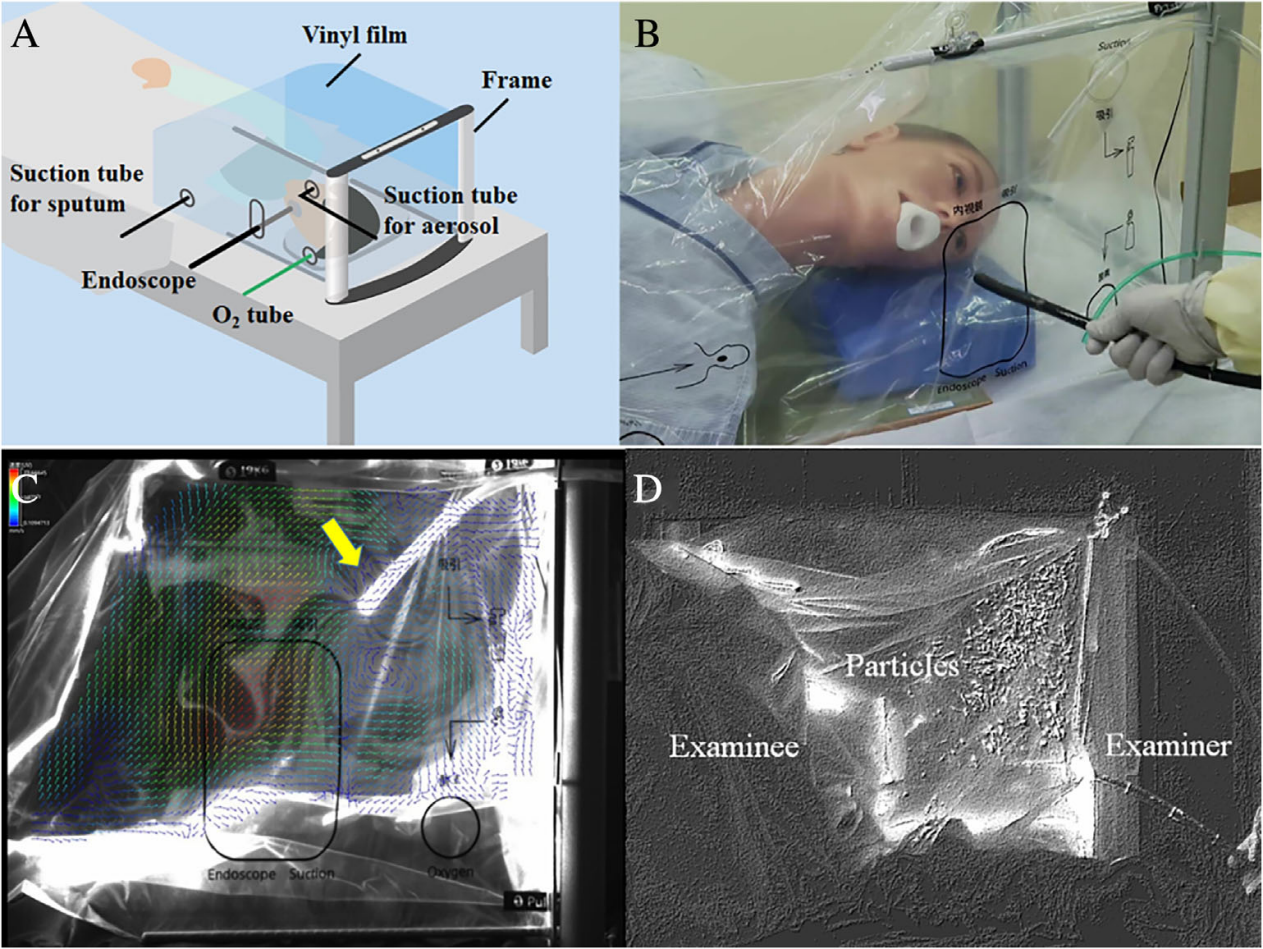
Patient‐covering negative pressure box. (a) Schema of concept model. (b) Photograph of Endo barrier. (c) Airflow vector flowing toward the continuous suction tube (yellow arrow). (d) Endo barrier in use: no particle scattering.

Prior to clinical application, we performed the following verification tests of the EB (Video S1, Supporting information). First, negative pressure was verified using a smoke test, and we confirmed the airflow vector was flowing toward the continuous suction tube (negative pressure: −40 kPa) inserted in the film (Fig. 1c). There was no smoke leakage out of the box. Second, we compared the visualized degree of particle (simulated aerosols) or food coloring (simulated droplets) scattering with and without the EB. The examiner and surrounding environment were exposed to particles and food coloring without EB use but not with it (Fig. 1d) in place. These results demonstrate the need for negative pressure and ability of the EB to reduce direct virus exposure for the examiner. In clinical practice, the EB worked well for patients, without hindering the scope of maneuver. In addition to the EB effects with very low risk of droplet or contact transmission, it takes another advantage of easy setup and disposal.

The EB may be an alternative for minimizing SARS‐CoV‐2 transmission during UGI.

## Supporting information

**Video S1.** This video presents the verification test of a patient‐covering negative pressure box for use during upper gastrointestinal endoscopy.
